# OsASA2 Loss‐of‐Function Confers Broad‐Spectrum Disease Resistance and Triggers Cell Death by Regulating Anthranilate Synthase Activity in Rice

**DOI:** 10.1111/mpp.70340

**Published:** 2026-09-06

**Authors:** Mingliang Guo, Shuyu Jiang, Ying Li, Zhiwei Chen, Shengping Li, Ziqiang Chen, Yuqing Niu, Mo Wang, Songbiao Chen, Dagang Tian

**Affiliations:** ^1^ Fujian Provincial Key Laboratory of Agricultural Genetic Engineering, Biotechnology Research Institute Institute of Crop Sciences (Fujian Germplasm Resources Center), Fujian Academy of Agricultural Sciences Fuzhou Fujian China; ^2^ College of Agriculture, College of Life Sciences Fujian Agriculture and Forestry University Fuzhou Fujian China; ^3^ Marine and Agricultural Biotechnology Laboratory Minjiang University Fuzhou China

**Keywords:** anthranilate synthase, basal resistance, lesion mimic mutant, *OsASA2*, signal transduction, Trp biosynthetic pathway

## Abstract

Lesion mimic mutants are valuable for studying plant cell death and defence mechanisms. Here, we identify a rice lesion mimic mutant, *osasa2‐1*, which exhibits spontaneous cell death and enhanced disease resistance. The mutant displays white spots on leaves that develop into large brown lesions. Histological analysis reveals increased cell death and H_2_O_2_ accumulation. Map‐based cloning identified the causal gene as *OsASA2* (LOC_Os03g15780). The *osasa2‐1* mutant carries a G496 deletion that introduces a premature stop codon, likely resulting in nonsense‐mediated decay of the mutant transcript. We demonstrate that OsASA2 interacts with OsASA1, OsASB1 and OsASB2. Its loss‐of‐function leads to hyperactivation of anthranilate synthase (AS) activity, enhancing tryptophan biosynthesis and metabolic flux into downstream pathways. The *osasa2‐1* mutant exhibits elevated reactive oxygen species (ROS) levels, increased callose deposition and upregulation of defence‐related genes, contributing to enhanced resistance against both the rice blast fungus *Magnaporthe oryzae* and the bacterial blight pathogen *Xanthomonas oryzae* pv. *oryzae*. Our findings highlight a novel regulatory role for *OsASA2* in conferring broad‐spectrum disease resistance by modulating AS activity and the tryptophan biosynthetic pathway in rice.

## Graphical abstract

Loss of OsASA2 disrupts anthranilate synthase regulation, triggering metabolic reprogramming, cell death and broad‐spectrum disease resistance in rice.

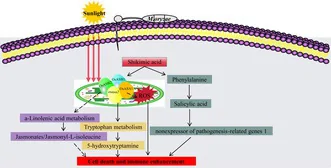

## 1 Introduction

Plants have evolved a two‐tiered immune system comprising pattern‐triggered immunity (PTI) and effector‐triggered immunity (ETI) to defend against pathogens (Schwessinger and Zipfel 2008; Faulkner and Robatzek 2012). ETI often involves a hypersensitive response (HR), characterized by rapid programmed cell death (PCD) at the infection site to restrict pathogen growth (Wu et al. 2014; Zebell and Dong 2015). This process features a burst of reactive oxygen species (ROS), HR‐mediated cell death, callose deposition, phytoalexin accumulation and upregulation of pathogenesis‐related (PR) genes (Yuan et al. 2021). Similar to pathogen‐induced HR, many plant lesion mimic mutants (LMMs) spontaneously develop defence‐related phenotypes in the absence of pathogens, leading to constitutive immune activation and significantly enhanced disease resistance (Sha et al. 2023). Functionally characterized rice LMMs include those affecting a heat‐stress transcription factor (SPL7; Yamanouchi et al. 2002), a translation elongation factor‐like protein (SPL33; Wang et al. 2017), two E3 ubiquitin ligase (OsCUL3a; Liu et al. 2017; SPL11, Zeng et al. 2004), a receptor‐like kinase (SPL36; Rao et al. 2021) and a porphyrin biosynthetic enzyme (RLIN1; Sun et al. 2011). These LMMs play pivotal roles in regulating disease resistance and PCD, contributing to broad‐spectrum resistance (Sun et al. 2024; Yan et al. 2022).

The involvement of jasmonic acid (JA) and salicylic acid (SA) signalling pathways in defence responses of rice LMMs is well documented (Tian et al. 2022). For instance, the rice cytochrome P450 gene *CYP71A1* (also known as *SL*), encoding tryptamine 5‐hydroxylase, converts tryptamine to serotonin. In the *sl‐MH‐1* mutant, lack of serotonin production leads to elevated ROS and SA levels, resulting in PCD and enhanced blast resistance (Fujiwara et al. 2010; Tian et al. 2020).

Anthranilate synthase (AS) is a key enzyme in the tryptophan (Trp) biosynthetic pathway in plants, composed of α‐ and β‐subunits (Tozawa et al. 2001). Trp levels are tightly regulated through feedback inhibition of AS activity. The α‐subunits catalyse the conversion of chorismate to anthranilate and contain a Trp‐binding site for feedback regulation. Although feedback‐insensitive ASα variants have been identified in several species (Tozawa et al. 2001; Bohlmann et al. 1996; Song et al. 1998), and critical residues for Trp binding have been characterized (Matsui et al. 1987; Caligiuri and Bauerle 1991; Graf et al. 1993), the detailed regulatory mechanisms of plant AS remain incompletely understood. In rice, the α‐subunits OsASA1 and OsASA2, along with the β‐subunits OsASB1 and OsASB2, form functional AS complexes (Zhang et al. 2001; Kanno et al. 2004; Inaba et al. 2007). While *OsASA2* expression is induced by chitin elicitors (Tozawa et al. 2001) and implicated in Trp metabolism during senescence (Kang et al. 2008), its specific role in linking primary metabolism to cell death and immune responses is unclear.

In this study, we identified a novel rice lesion mimic mutant, *osasa2‐1*, from a tissue culture‐regenerated population. Unlike previously reported OsASA2‐related mutants that primarily exhibit altered Trp accumulation, *osasa2‐1* displays a strong cell death phenotype and enhanced resistance to both *Magnaporthe oryzae* and *Xanthomonas oryzae* pv. *oryzae* (Xoo). We demonstrate that loss of *OsASA2* function disrupts the regulatory AS complex, leading to (1) hyperactivation of AS activity and metabolic reprogramming of the Trp pathway; (2) accumulation of ROS and induction of PCD; and (3) activation of SA and JA signalling pathways, culminating in broad‐spectrum disease resistance without a significant penalty to agronomic traits.

## 2 Results

### 2.1 The *osasa2‐1* Mutant Exhibits Spontaneous Lesions and Enhanced Immune Responses

**FIGURE 1:**
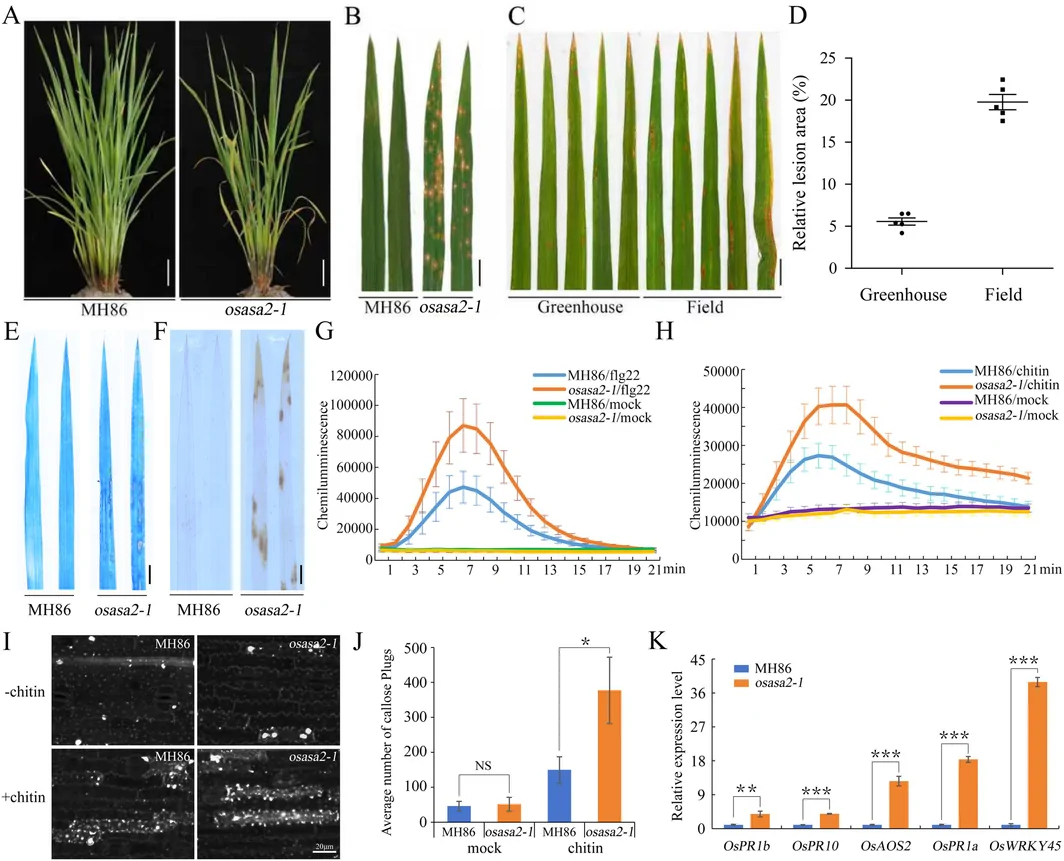
Phenotypic characterization of the *osasa2‐1* mutant. (A) Photographs of the upper leaves of the main stem of *osasa2‐1* mutant and wild‐type Minghui86 (MH86) plants grown in the field, taken at the tillering stage. The *osasa2‐1* mutant exhibited reddish‐brown lesions scattered across its leaves, which appeared on older leaves at the late tillering stage and gradually spread to the entire leaves. Bar = 3 cm. (B) The upper leaves of the main stem from field‐grown *osasa2‐1* mutant and MH86 wild‐type plants at the late tillering stage. Bar = 1 cm. (C) Upper leaves from the main stem of field‐grown (left) and greenhouse‐grown (right) *osasa2‐1* mutant and MH86 wild‐type plants at the early tillering stage. Bar = 1 cm. Leaves were photographed after detachment. (D) Lesion areas on *osasa2‐1* leaves were smaller in greenhouse‐grown plants than in field‐grown plants at the same early tillering stage, accounting for the observed difference in lesion intensity. (E) Trypan blue staining of the 5‐week‐old *osasa2‐1* mutant and MH86 leaves. (F) 3,3′‐diaminobenzidine (DAB) staining of the 5‐week‐old *osasa2‐1* mutant and MH86 leaves. (G) Reactive oxygen species (ROS) accumulation dynamics in *osasa2‐1* mutant and MH86 plants after 400 nM flg22 and water (mock) treatments. Error bars represent the SE; *n* = 3. (H) ROS accumulation dynamics in *osasa2‐1* mutant and MH86 plants after 500 nM chitin and water (mock) treatments. Error bars represent the SE; *n* = 3. (I) Callose deposition in *osasa2‐1* mutant and MH86 plants after 800 nM chitin treatment. (J) Quantification of the callose deposition in *osasa2‐1* mutant and MH86 plants per field of view by ImageJ. Error bars indicate SE; *n* = 3. Student's *t*‐test; *p < 0.05; NS, not significant. (K) Reverse transcription‐quantitative PCR was used to analyse the expression of *OsPr1b, OsPR10, OsAOS2, OsPR1a*, and *OsWRKY45*. Data were normalized to the expression level of the constitutively expressed *OsACTIN* gene. Error bars represent the SE; *n* = 3 (Student's *t*‐test was conducted between *osasa2‐1* and MH86; *p < 0.05, **p < 0.01, ***p < 0.001).

The *osasa2‐1* mutant was identified from an M_2_ population of tissue culture‐derived mutants of the rice cultivar Minghui86 (MH86). Under summer field conditions, *osasa2‐1* developed sporadic reddish‐brown lesions on leaves, starting on older leaves at the late tillering stage and progressing to entire leaves (Figure 1A,B). In greenhouse conditions, lesions appeared later (heading stage) and were smaller and fewer than in the field, suggesting environmental modulation (e.g., by light and temperature) of the cell death phenotype (Figure 1C,D). Plant growth and agronomic traits were largely unaffected (Table S1).

Trypan blue and 3,3′‐diaminobenzidine (DAB) staining revealed significantly more dead tissue and higher H_2_O_2_ accumulation in *osasa2‐1* leaves compared to MH86 (Figure 1E,F). The mutant also showed a faster and stronger ROS burst in response to flg22 and chitin treatments (Figure 1G,H) and enhanced chitin‐induced callose deposition (Figure 1I,J), indicating a potentiated PTI response. Reverse transcription‐quantitative PCR (RT‐qPCR) analysis showed significant upregulation of SA pathway‐related genes (*OsPR1a*, *OsPR1b*, *OsPR10* and *OsWRKY45*) and a 12‐fold increase in the expression of *OsAOS2*, a key JA biosynthetic gene (Figure 1K). These data suggest that loss of *OsASA2* function enhances basal defence and activates SA/JA signalling.

### 2.2 *OsASA2* Encodes an Anthranilate Synthase α‐Subunit and Is Responsible for the Mutant Phenotype

**FIGURE 2:**
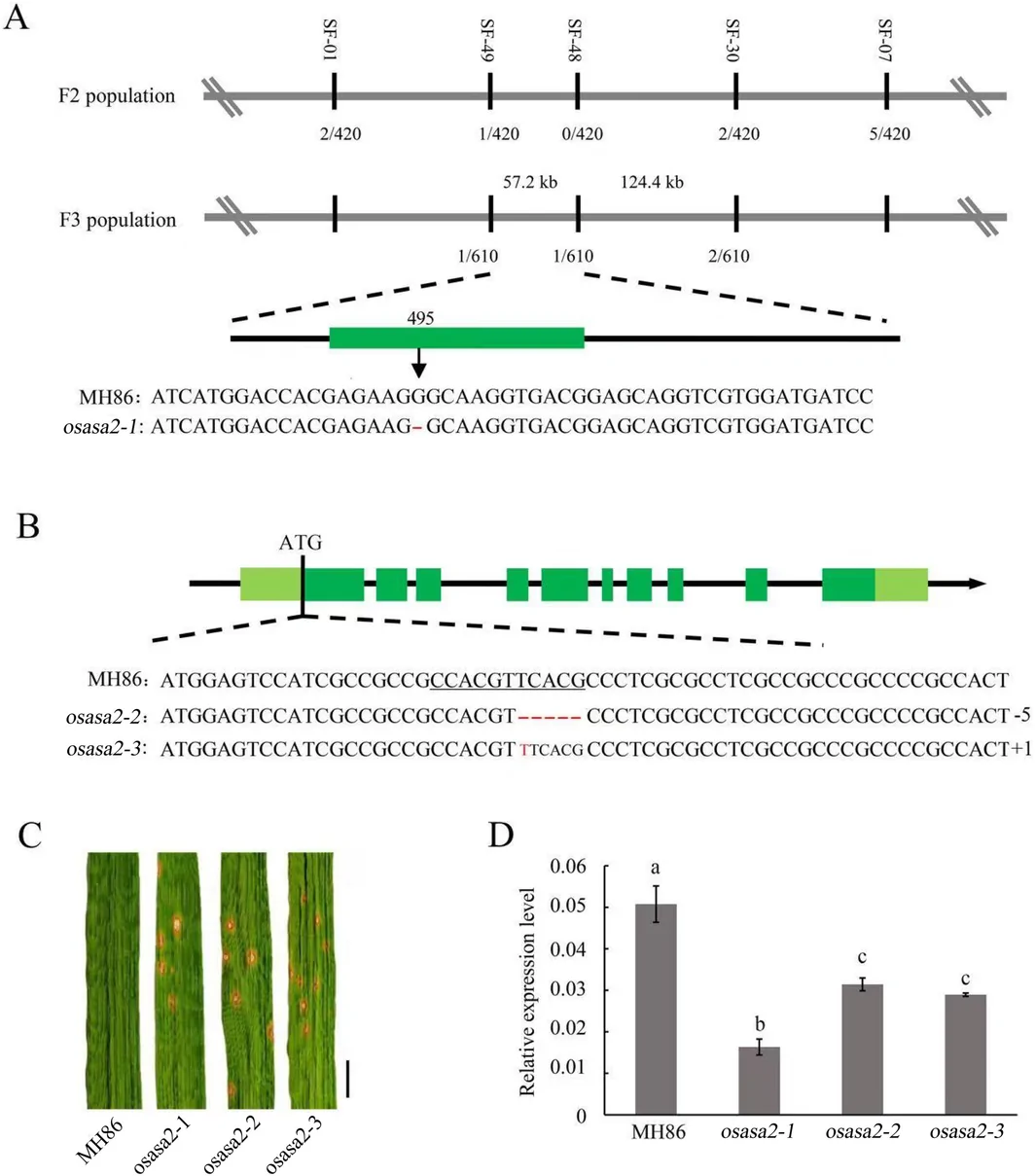
Map‐based cloning of *OsASA2*. (A) Delimitation of the candidate genomic region of *OsASA2*. (B) Mutational sites of mutants *osasa2‐2* and *osasa2‐3*. (C) Leaves (second from the top) of mutants *osasa2‐1, osasa2‐2* and *osasa2‐3* plants at tillering stage. Bar = 1 cm (D) Transcription levels of *OsASA2* gene in Minghui86 (MH86) and *osasa2‐1, osasa2‐2* and *osasa2‐3*. Error bars represent the SE; *n* = 3. Data are presented as the means of three independent replicates ± SD and were analysed by one‐way ANOVA followed by Tukey's honestly significant difference (HSD) post hoc test (α = 0.05) to determine pairwise differences among groups. Bars with different letters are significantly different from each other.

Genetic analysis indicated the *osasa2‐1* phenotype was controlled by a single recessive nuclear gene (normal:lesion mimic = 1216:420, χ^2^ [3:1] = 0.394 < χ^2^ 0.05 = 3.84; *p* > 0.05). Bulked segregant analysis mapped the locus to a 57.2 kb interval on chromosome 3 (Figure 2A). The 57.2‐kb interval contains 11 predicted ORFs. Sequencing identified a 1‐bp deletion in the second exon of LOC_Os03g15780 (Figure 2B), which encodes anthranilate synthase component I‐1, previously named *OsASA2* (Tozawa et al. 2001). This deletion causes a premature stop codon (Table S2). The other 10 genes are either transposon‐related or of unknown function, and none showed sequence polymorphisms between the mutant and wild type. Two additional CRISPR/Cas9‐generated allelic genes, *osasa2‐2* and *osasa2‐3*, carrying different frame‐shift mutations in the first exon (Figure 2B; Table S2), recapitulated the lesion mimic phenotype (Figure 2C) and had reduced *OsASA2* expression (Figure 2D). Furthermore, the cell death phenotype was rescued by reintroducing the wild‐type *OsASA2* genomic fragment (Figure S1), confirming that mutation in *OsASA2* causes the observed phenotypes.

### 2.3 OsASA2 Interacts With OsASA1, OsASB1 and OsASB2 in Chloroplasts

**FIGURE 3:**
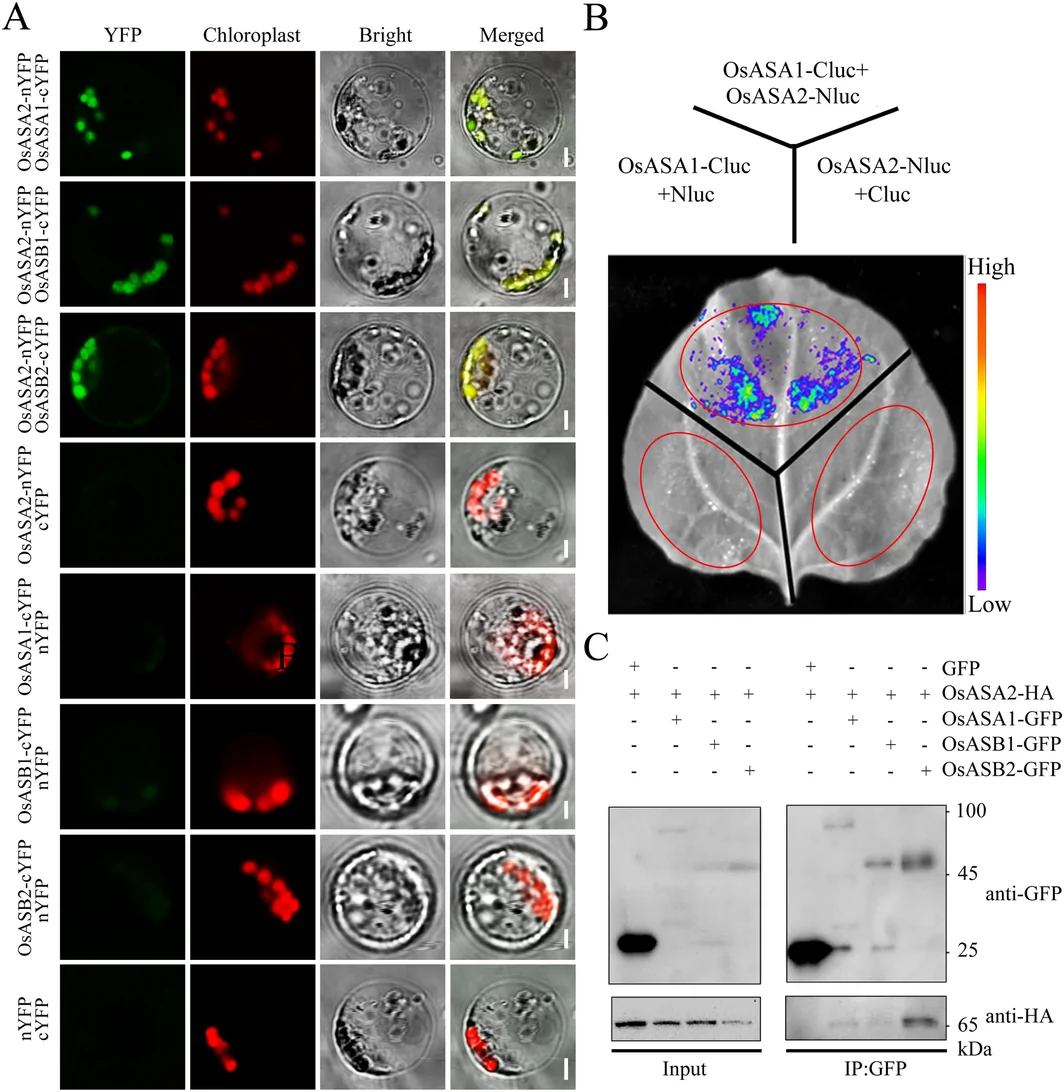
OsASA2 interacts with other anthranilate synthase subunits in vivo. (A) Bimolecular fluorescence complementation assays showing the interaction between OsASA2 and OsASA1, OsASB1, OsASB2 in rice. The OsASA2 and OsASA1, OsASB1, OsASB2 proteins were fused to the VN‐ or VC‐terminal fragment of pRTVcVN and transiently expressed in rice. The YFP signal was evaluated via confocal microscopy at 3 days after infiltration. Bar = 50 μm. (B) Interaction between OsASA2 and OsASA1 detected with firefly luciferase complementation imaging assay in plants. The protein interaction was indicated by fluorescence signal. The colour bar on the right represents the range of luminescence intensity in the image. The red circle is the injection area of *Agrobacterium tumefaciens* . (C) Immunoprecipitation assay proved that OsASA2 interacts with OsASA1, OsASB1 and OsASB2 in vivo. OsASA1, OsASB1 and OsASB2 proteins were immunoprecipitated by GFP antibody, followed by immunoblot analysis with anti‐HA antibody.

Rice AS comprises two α‐subunit isozymes (OsASA1 and OsASA2) and two β‐subunit isozymes (OsASB1 and OsASB2) (Kanno et al. 2004). All four proteins localized to chloroplasts (Figure S2). Bimolecular fluorescence complementation (BiFC) assays in rice protoplasts confirmed that OsASA2 interacted with OsASA1, OsASB1 and OsASB2, with fluorescence signals co‐localized with chloroplasts (Figure 3A). A firefly luciferase complementation imaging (LCI) assay in *Nicotiana benthamiana* leaves showed strong interaction between OsASA2 and OsASA1 (Figure 3B). Co‐immunoprecipitation (Co‐IP) assays in rice protoplasts further validated these interactions in vivo (Figure 3C). These results establish that OsASA2 forms a complex with other AS subunits in chloroplasts.

### 2.4 Loss of OsASA2 Function Reprogrammes Tryptophan Metabolism

**FIGURE 4:**
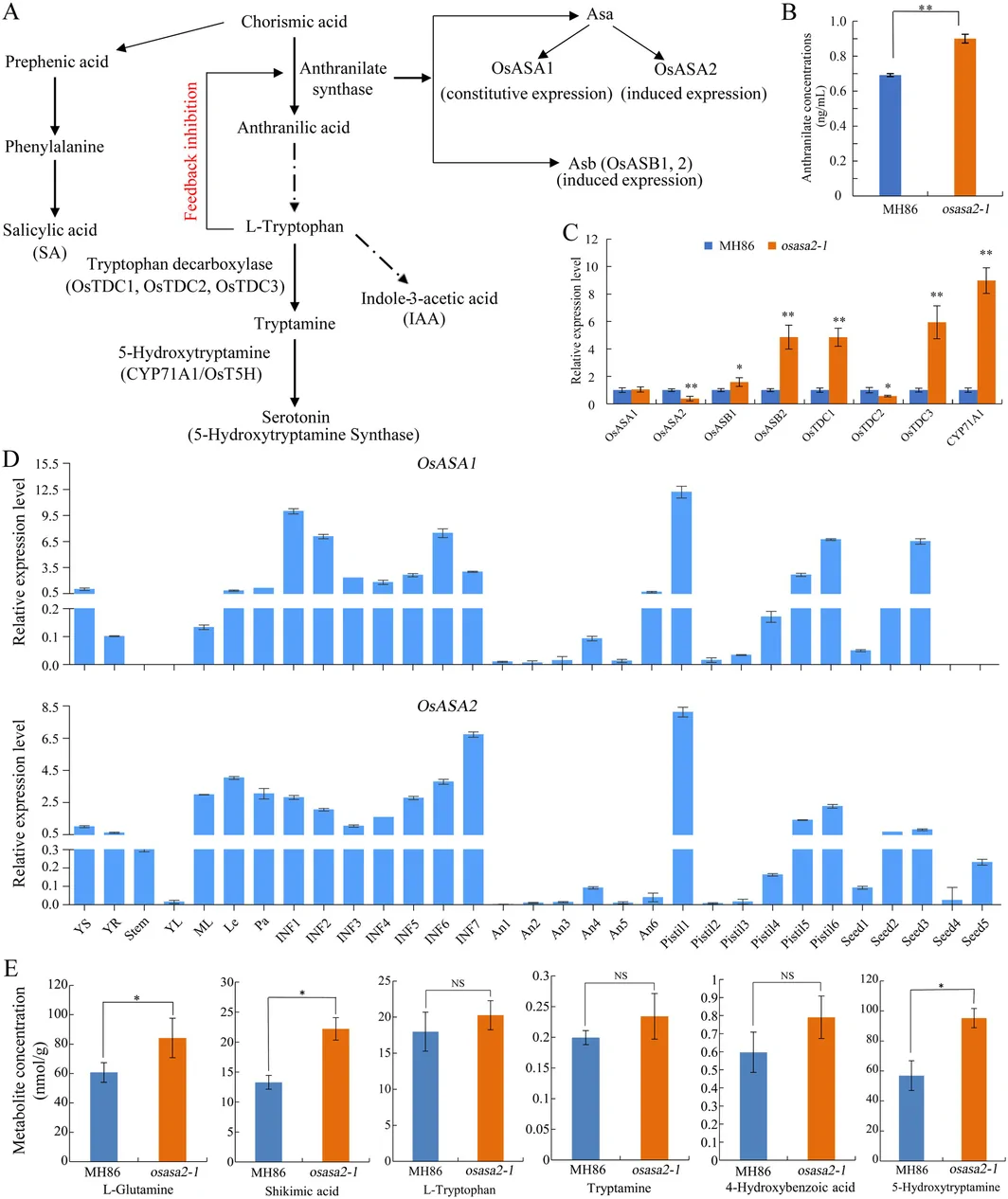
Loss of *OsASA2* reprogrammes tryptophan metabolism. (A) The tryptophan metabolic process. (B) The concentrations of anthranilate in vitro anthranilate synthase (AS) assay in rice seedlings of Minghui86 (MH86) and *osasa2‐1*, as detected by UPLC‐MS/MS. Means ± SE represent three independent biological replicates. Error bars represent the SE; *n* = 3 (*t*‐test; **p < 0.01). (C) Relative expression levels of genes in tryptophan metabolism between *osasa2‐1* mutant and wild‐type MH86 at 48 h post‐inoculation (hpi). The expression level of MH86 was set to 1. Error bars represent the SE; *n* = 3 (*t*‐test; *p < 0.05, **p < 0.01). (D) Expression patterns of *OsASA1* and *OsASA2* relative to *actin*. YS, 10‐day‐old seedling; YR, the root of YS; Stem, the stem on heading date; YL, the leaf of YS; ML, the leaf of mature plants; Le and Pa, the lemma and palea of the spikelet before pollination; INF1, 0–1 cm inflorescence; INF2, 2–3 cm inflorescence; INF3, 3–5 cm inflorescence; INF4, 5–10 cm inflorescence; INF5, 10–15 cm inflorescence; INF6, 15–20 cm inflorescence; INF7, 20–25 cm inflorescence; An1 and Ov1, the anther and pistil of 2–3 mm spikelet; An2 and Ov2, the anther and pistil of 3–4 mm spikelet; An3 and Ov3, the anther and pistil of 4–5 mm spikelet; An4 and Ov4, the anther and pistil of 5–6 mm spikelet; An5 and Ov5, the anther and pistil of 6–7 mm spikelet; An6 and Ov6, the anther and pistil of the spikelet before pollination; Seed1‐5, the seeds of 1, 3, 7, 10 and 25 days after pollination. (E) Levels of L‐glutamine, shikimic acid, L‐tryptophan, tryptamine, 4‐hydroxybenzoic acid and 5‐hydroxytryptamine in the leaves of 6‐week‐old wild‐type MH86 and mutant *osasa2‐1*. Error bars represent the SE; *n* = 3 (*t*‐test; *p < 0.05).

AS catalyses the conversion of chorismate to anthranilate, the first committed step in Trp biosynthesis (Figure 4A). We investigated the metabolic consequences of *OsASA2* disruption. An in vitro AS activity assay showed a significant increase in anthranilate production in *osasa2‐1* extracts compared to MH86 (Figure 4B, Figure S3), indicating hyperactive AS activity in the mutant.

Gene expression analysis revealed that loss of *OsASA2* function led to marked upregulation of several genes in the Trp pathway, including the β‐subunit genes *OsASB1* and *OsASB2*, tryptophan decarboxylase genes *OsTDC1* and *OsTDC3*, and the serotonin synthesis gene *CYP71A1* (Figure 4C). *OsASA1* transcript levels showed no significant change under basal conditions (Figure 4C), but expression profiling across tissues indicated that *OsASA1* and *OsASA2* had similar and complementary expression patterns, particularly in mature leaves (Figure 4D). Metabolite profiling confirmed elevated levels of pathway intermediates and derivatives (L‐glutamine, shikimic acid, L‐tryptophan, tryptamine, 4‐hydroxybenzoic acid and 5‐hydroxytryptamine [serotonin]) in *osasa2‐1* leaves with lesions, with L‐glutamine, shikimic acid and serotonin showing the most pronounced accumulation (Figure 4E). This indicates a global reprogramming of Trp metabolism upon *OsASA2* disruption.

### 2.5 *OsASA1* Is Not the Primary Determinant of the Lesion Mimic Phenotype

Given the homology between *OsASA1* and *OsASA2*, we investigated if loss of *OsASA1* could induce similar phenotypes. We generated two independent CRISPR‐mediated knockout mutant lines of *OsASA1*. One line carries a 9‐bp deletion at target site 1 and a G base deletion at target site 2. An additional mutant allele features a GG base deletion at target site 1. All mutations within the *OsASA1* coding sequence are predicted to trigger premature translation termination (Table S2). The two mutant lines did not exhibit spontaneous lesions (Figure S4A) or enhanced *M. oryzae* (Figure S4B–F) and Xoo (Figure S4G,H) resistance, demonstrating that *OsASA1* deficiency alone is insufficient to trigger cell death or immunity. This aligns with recent findings where mutation in *OsASA2* (also called *ELS1*) triggers a genetic compensation response involving *OsASA1* upregulation, which hyperactivates the Trp pathway leading to ROS and PCD (Li 2024). Our failure to obtain *osasa1 osasa2* double mutants suggests simultaneous disruption of both α‐subunit genes is lethal, underscoring their essential and complementary functions.

### 2.6 Transcriptomic and Proteomic Analyses Reveal Activation of Defence and Metabolic Pathways

**FIGURE 5:**
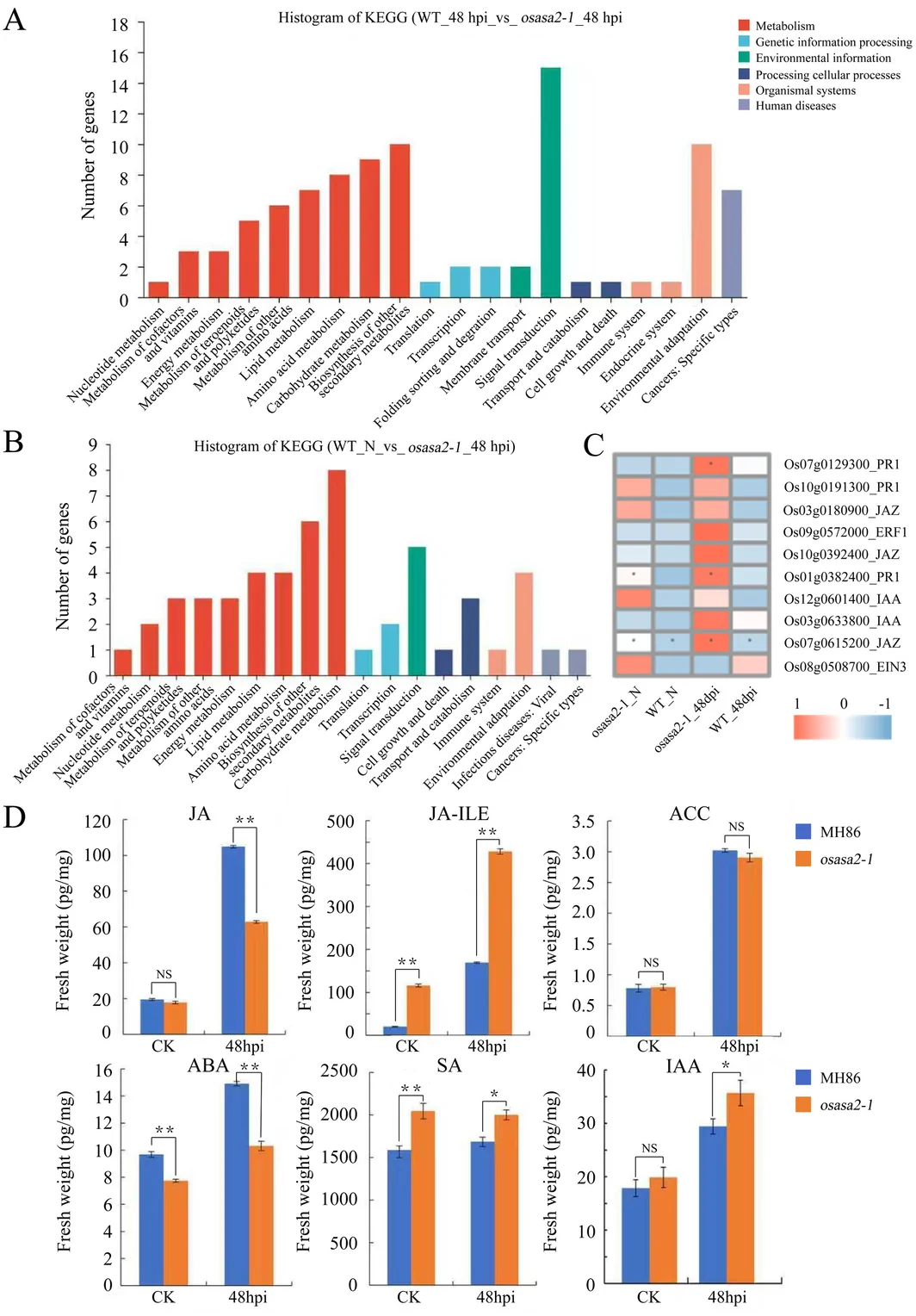
Activation of signalling pathways in *osasa2‐1* mutant. (A, B) KEGG annotation analysis of differential expressed genes in *osasa2‐1* mutant and wild type (WT) at 48 h post‐inoculation (hpi), and *osasa2‐1* mutant and WT under cell death. N indicates naturally occurring lesion‐mimic. (C) Heatmap of 10 genes of the contrasting expression between four groups, *osasa2‐1*_N and WT_N, WT and *osasa2‐1* mutant under cell death, *osasa2‐1*_48 hpi and WT_48 hpi, WT and *osasa2‐1* mutant at 48 hpi with *Magnaporthe oryzae* . The heat‐map colour scale is shown to the right of each panel showing log_2_ fold‐change (mutant/WT) of transcript abundance (RNA‐seq, *n* = 3). (D) Contrasting expression of phytohormones jasmonic acid (JA), jasmonate‐isoleucine (JA‐Ile), salicylic acid (SA), abscisic acid (ABA), indole‐3‐acetic acid (IAA) and 1‐aminocyclopropane‐1‐carboxylic acid (ACC) between the *osasa2‐1* mutant and wild‐type plants at 0 (CK) and 48 hpi with *M. oryzae*. Error bars represent the SE; *n* = 3 (*t*‐test; *p < 0.05, **p < 0.01).

RNA‐seq analysis of *osasa2‐1* under mock treatment, *M. oryzae* infection (48 hpi), and spontaneous cell death conditions revealed distinct transcriptional profiles (Figures S5 and S6). Kyoto Encyclopedia of Genes and Genomes (KEGG) enrichment analysis highlighted ‘signal transduction’ as the most prominently activated pathway in infected and lesion‐forming tissues compared to mock treatment (Figure 5A,B; Figure S7). Numerous upregulated genes were associated with auxin, ethylene, JA and SA signalling pathways (Figure 5C; Figure S8).

**FIGURE 6:**
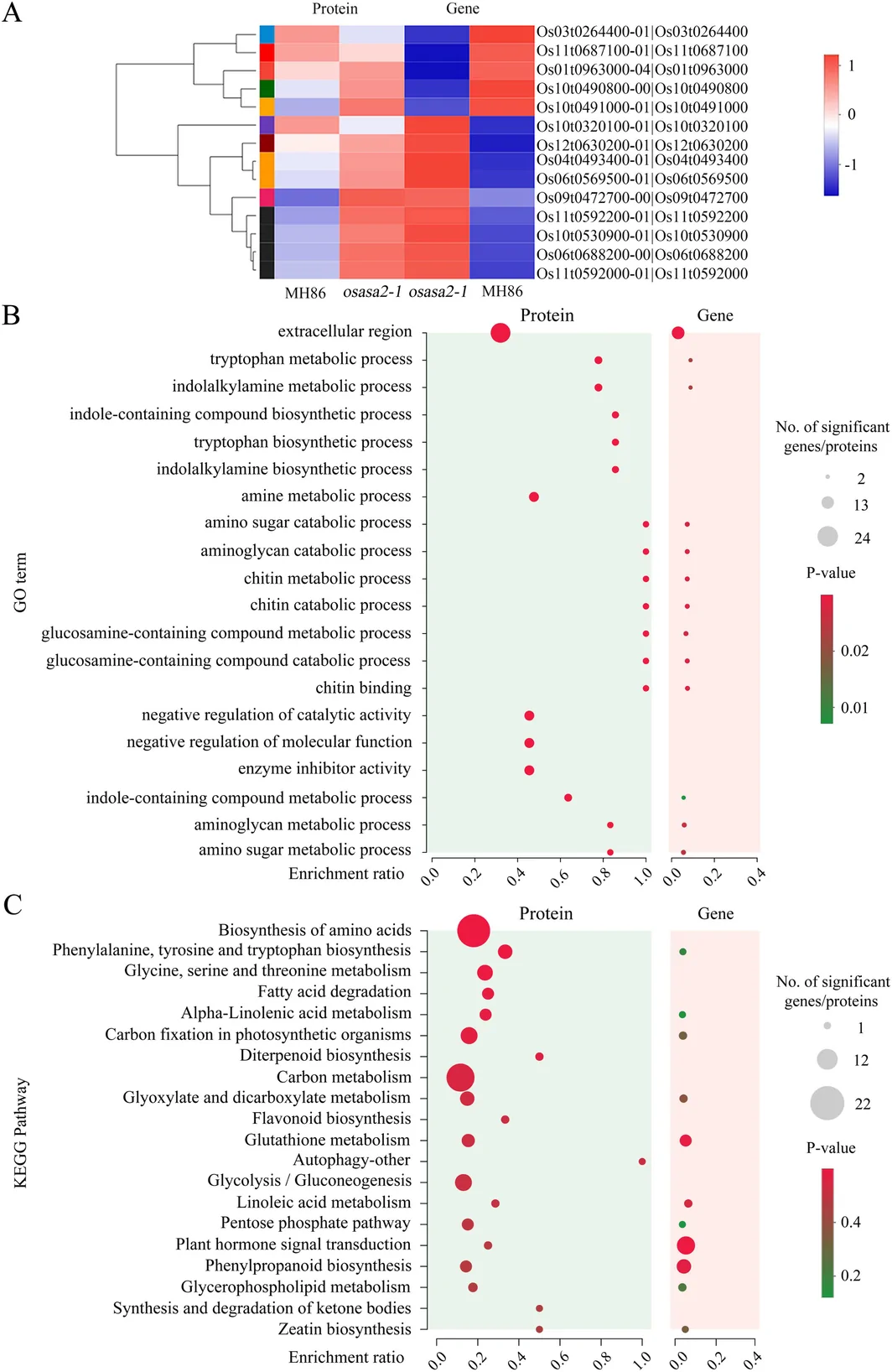
Integrated transcriptomic and proteomic analysis. (A) Common differentially expressed genes and proteins in the *osasa2‐1* mutant under the cell death conditions compared with wild‐type (Minghui86, MH86) plants. This figure displays a heatmap representing protein/gene expression data. Each column corresponds to a sample group, while each row represents an individual protein or gene. Colour intensity reflects relative expression levels, with the specific numerical scale indicated in the colour bar at the upper left. The left dendrogram illustrates hierarchical clustering of proteins/genes; branch proximity indicates similarity in expression profiles. The top dendrogram represents sample clustering, where closer branches denote more similar overall expression patterns across all proteins/genes. (B) and (C) GO and KEGG enrichment analysis of integrated differentially expressed genes and proteins in the *osasa2‐1* mutant exhibiting cell death compared with wild‐type plants. The vertical axis represents GO terms; the horizontal axis represents enrichment rate, calculated as: num_in_study/num_in_pop. Each bubble in the figure represents a GO subcategory. The size of the bubble is proportional to the number of proteins or genes enriched in that GO subcategory. The colour of the bubble represents the *p*‐value.

Integrative analysis of iTRAQ‐based proteomics (Figures S9 and S10) and RNA‐seq data from tissues undergoing cell death showed a low overall correlation but identified 14 genes/proteins with consistent directional changes (Figure 6A; Table S3; Figure S11). GO and KEGG enrichment analyses of these integrated datasets highlighted processes including ‘tryptophan metabolic process,’ ‘indolalkylamine metabolic process’, ‘α‐linolenic acid metabolism’ and ‘phenylalanine, tyrosine and tryptophan biosynthesis’ (Figure 6B,C; Figure S12). This confirms the activation of Trp and related metabolic pathways at both transcriptional and translational levels in the mutant.

### 2.7 Altered Phytohormone Profiles in the *osasa2‐1* Mutant

Targeted hormone profiling revealed complex changes in *osasa2‐1*. While SA and JA‐Ile levels increased in both genotypes upon infection, *osasa2‐1* had significantly higher JA‐Ile but lower free JA levels compared to MH86 at 48 hpi (Figure 5D). Abscisic acid (ABA) levels decreased upon infection and were lower in *osasa2‐1* than in MH86. Levels of 1‐aminocyclopropane‐1‐carboxylic acid (ACC, the ethylene precursor) showed no significant difference between the mutant and wild‐type after infection (Figure 5D). This indicates a specific reprogramming of SA and active JA (JA‐Ile) signalling in the mutant.

### 2.8 *OsASA2* Loss‐Of‐Function Confers Broad‐Spectrum Disease Resistance

**FIGURE 7:**
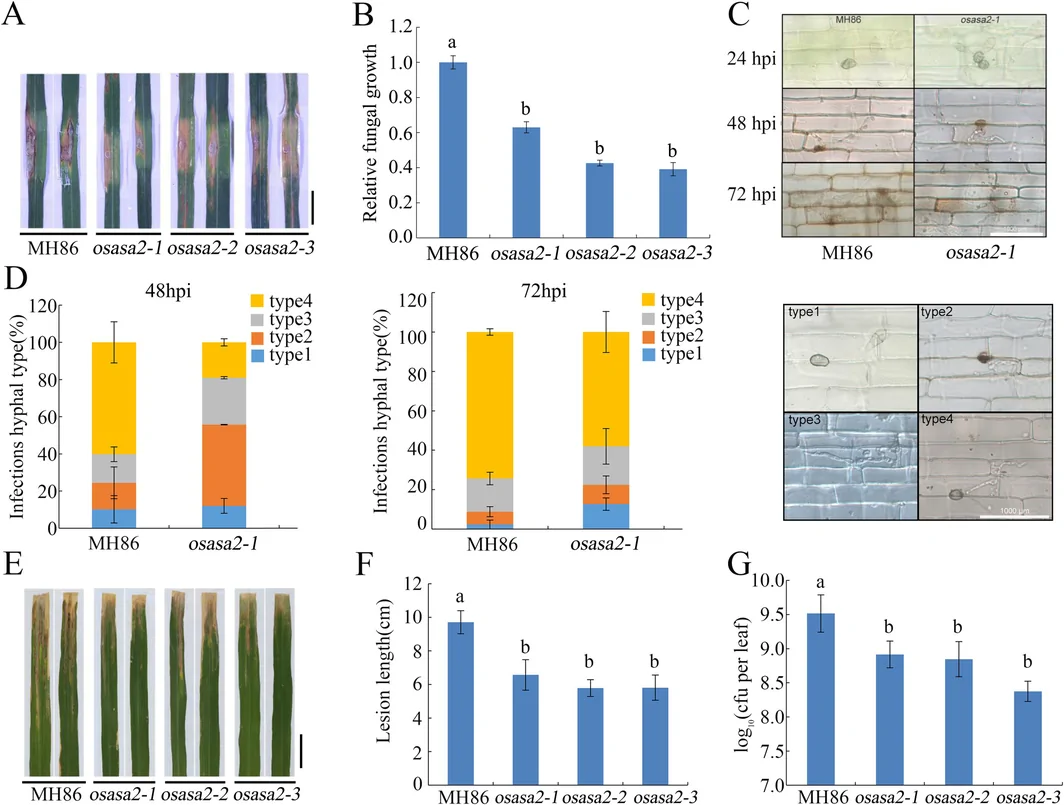
*osasa2‐1* confers enhanced resistance to *Magnaporthe oryzae* and *Xanthomonas oryzae* pv. *oryzae* (Xoo). (A) Phenotype of wild‐type Minghui86 (MH86) and mutants *osasa2‐1, osasa2‐2* and *osasa2‐3* punched with *M. oryzae* conidia. Bar = 2 cm. (B) Relative fungal growth of MH86 and mutants *osasa2‐1*, *osasa2‐2* and *osasa2‐3* at 48 h post‐inoculation (hpi) with the compatible *M. oryzae* FJ‐1. Data are presented as the means of three independent replicates ± SD and were statistically analysed by one‐way ANOVA followed by Tukey's honestly significant difference (HSD) post hoc test (*α* = 0.05) to determine pairwise differences among groups. Bars with different letters are significantly different from each other. Error bars represent the SE; *n* = 3. (C) Comparative analysis of invasive growth of *M. oryzae* FJ‐1 in leaf sheathes of wild‐type MH86 and mutant *osasa2‐1* at 24, 48 and 72 hpi. The infected cells with a microscope under bright field. Bar = 1000 μm. (D) Statistical analysis of invasive growth of FJ‐1 hyphae in leaf sheath cells of wild‐type MH86 and mutant *osasa2‐1* at 48 and 72 hpi. 100 appressorium infiltration sites were counted. The invasive hyphae were of type 1 to type 4 (type 1, only appressorium. Type 2, forming 1 infection hypha. Type 3, infectious hypha begin to expand but remain confined to the initial infected cell. Type 4, the hypha spreads to neighbouring cells). The leaf sheath infection experiment was repeated more than three times with similar results. Bar = 1000 μm. They are from the same set of inoculated leaves at 12 days post‐inoculation. Error bar represent the SE; *n* = 3. (E) Phenotype of wild‐type MH86 and mutants *osasa2‐1*, *osasa2‐2* and *osasa2‐3* inoculated with Xoo. Bar = 2 cm. (F) Blight lesion lengths on leaves of mutants *osasa2‐1*, *osasa2‐2*, *osasa2‐3* and MH86 were measured. Data are presented as the means of three independent replicates ± SE and were analysed by one‐way ANOVA followed by Tukey's honestly significant difference (HSD) post hoc test (*α* = 0.05) to determine pairwise differences among groups. Bars with different letters are significantly different from each other. Error bar represent the SE; *n* = 3. (G) Xoo bacterial populations were counted. Cfu means colony‐forming units. Data are presented as the means of three independent replicates ±SD and were analysed by one‐way ANOVA followed by Tukey's honestly significant difference (HSD) post hoc test (*α* = 0.05) to determine pairwise differences among groups. Bars with different letters are significantly different from each other. Error bar represent the SE; *n* = 3.

Based on the enhanced immune responses, we evaluated resistance against major rice pathogens. MH86 (wild‐type) and *osasa2‐1* mutant seedlings were planted in a field nursery subjected to natural *M. oryzae* infection. Following 1 month of natural infection, the *osasa2‐1* showed markedly elevated blast resistance (Figure S13). Punch‐inoculation with *M. oryzae* (strain FJ‐1) resulted in significantly smaller lesions and lower fungal biomass in *osasa2‐1* and its alleles (*osasa2‐2* and *osasa2‐3*) compared to MH86 (Figure 7A,B). Leaf sheath inoculation assays showed that bright‐field images of invasive hyphae (IH) in leaf sheaths of *M. oryzae* were largely confined to the initially infected cell in *osasa2‐1* at 48 and 72 hpi, whereas they spread to neighbouring cells in MH86 (Figure 7C). Furthermore, there was more extensive cell death associated with IH restriction in the mutant (Figure 7D). Similarly, infection with Xoo (isolate PXO99) led to significantly shorter lesion lengths and lower bacterial populations in *osasa2‐1*, *osasa2‐2* and *osasa2‐3* compared to MH86 (Figure 7E–G). These results demonstrate that *OsASA2* loss‐of‐function confers enhanced resistance to both fungal and bacterial pathogens.

## 3 Discussion

### 3.1 OsASA2 Is a Central Modulator of the AS Complex and Trp Metabolism

Trp is a precursor for auxin and various defence compounds. In rice, two AS α‐subunit genes, *OsASA1* and *OsASA2*, exist (Kanno et al. 2004). Our study revealed that OsASA2 interacts with OsASA1 and the β‐subunits OsASB1/OsASB2 within chloroplasts, forming a regulatory complex. The loss of *OsASA2* eliminates one α‐subunit, but the remaining *OsASA1*‐containing complex shows altered feedback regulation, leading to a net increase in Trp pathway flux in vivo despite lower total enzyme activity. This is supported by elevated AS activity in vitro, upregulation of β‐subunit and downstream genes (*OsASB1*, *OsASB2*, *OsTDC1*, *OsTDC3* and *CYP71A1*), and accumulation of multiple pathway metabolites (Figure 4). This paradoxical increase in activity upon loss of one α‐subunit suggests *OsASA2* plays a critical modulatory, possibly inhibitory, role within the complex, fine‐tuning Trp biosynthesis. Our findings provide new insights into the complex regulation of AS in plants and identify key interaction nodes that could be targeted to manipulate Trp metabolism for improved disease resistance.

### 3.2 Metabolic Reprogramming Links Trp Biosynthesis to Cell Death and Immune Signalling

The hyperactive Trp metabolism in *osasa2‐1* leads to an overflow of intermediates into branching pathways, the increase in shikimic acid and phenylalanine is likely due to a general upregulation of the shikimate pathway in response to reduced anthranilate synthase activity, as a compensatory mechanism. Chorismate, the substrate for AS, can also be diverted into the SA biosynthesis pathway (Zhu et al. 2025; Liu et al. 2025). The significant accumulation of SA and the active JA conjugate JA‐Ile in *osasa2‐1* (Figure 5D), alongside elevated serotonin (Figure 4E), points to a metabolic rewiring that fuels defence signalling molecules. The resulting ROS burst, likely originating from multiple sources including tryptamine oxidation and JA signalling potentiation, triggers PCD, manifesting as lesions. This cell death, in turn, reinforces defence by restricting pathogen spread, as seen in the *M. oryzae* infection assays (Figure 7C,D). Our model integrates primary metabolism (Trp pathway) with defence hormone synthesis (SA and JA) and execution mechanisms (ROS, PCD), explaining how a lesion mimic phenotype translates into broad‐spectrum resistance.

### 3.3 Synergistic Action of Multiple Hormones Underlies the Resistance Phenotype

The *osasa2‐1* mutant exhibits a coordinated activation of multiple defence pathways. The upregulation of SA‐ and JA‐responsive genes (Figures 1M and 5C), along with altered hormone profiles (high SA and high JA‐Ile), indicates a synergistic interaction between these signalling sectors. This is reminiscent of our previous work on the *sl* mutant, where disrupted serotonin biosynthesis also led to SA/JA accumulation and resistance (Tian et al. 2020, 2022). The current study positions *OsASA2* upstream of such hormonal cross‐talk. Notably, while transcripts for auxin‐ and ethylene‐related genes were upregulated (Figures 5C and 6A), free ACC levels did not differ significantly from the wild type upon infection (Figure 5D), highlighting the specificity and complexity of the hormonal network orchestrated by *OsASA2*. This multifaceted signalling output likely contributes to the robust and broad‐spectrum resistance observed.

### 3.4 Perspectives and Working Model

**FIGURE 8:**
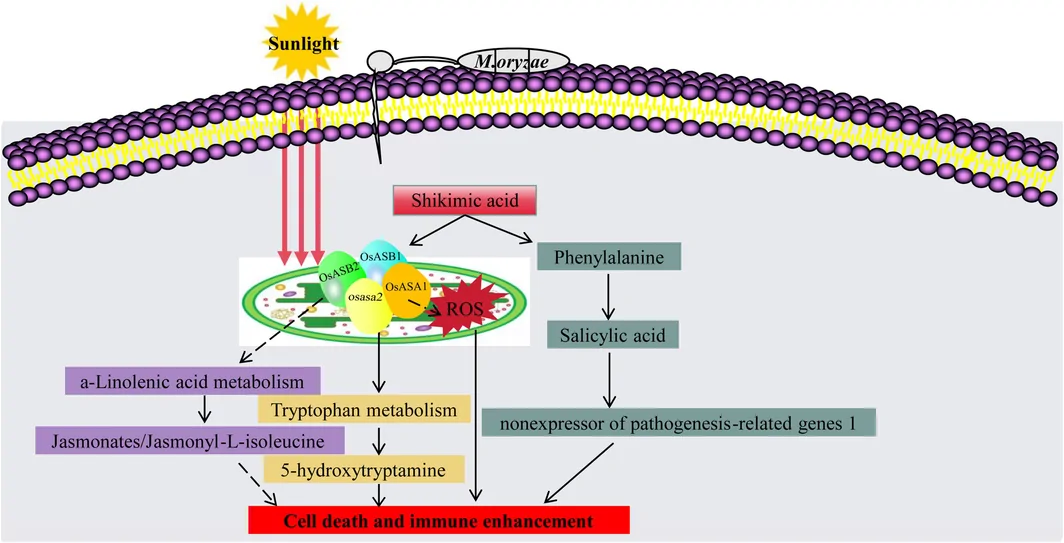
Working model for OsASA2‐mediated regulation of cell death and immunity. In the wild type, the OsASA2‐containing anthranilate synthase (AS) complex maintains regulated tryptophan (Trp) biosynthesis. In the *osasa2‐1* mutant, loss of OsASA2 disrupts complex regulation, leading to hyperactive AS (involving compensatory OsASA1). This causes metabolic overflow, increasing flux towards salicylic acid, jasmonic acid and serotonin biosynthesis. The accumulation of these defence signals, along with elevated reactive oxygne species (ROS), triggers programmed cell death (PCD), which manifests as lesions and restricts pathogen spread, thereby conferring broad‐spectrum disease resistance. Solid arrows indicate direct conversions or activations; dashed arrows indicate indirect or multistep processes.

Based on our findings, we propose a working model (Figure 8). In wild‐type plants, OsASA2 interacts with OsASA1, OsASB1 and OsASB2 to form a properly regulated AS complex, maintaining Trp homeostasis and preventing metabolic overflow into defence hormone pathways. In the *osasa2‐1* mutant, the loss of *OsASA2* disrupts this regulation, leading to compensatory upregulation and hyperactivity of the remaining AS complex (OsASA1‐ASB1/2). This drives excessive flux through the Trp pathway, diverting chorismate and pathway intermediates towards the biosynthesis of SA, JA precursors and serotonin. The accumulation of these signalling molecules, particularly SA and JA‐Ile, along with ROS derived from perturbed metabolism, initiates and amplifies PCD. This cell death response forms the basis of the lesion mimic phenotype and concurrently establishes a potentiated immune state, conferring enhanced resistance against diverse pathogens like *M. oryzae* and Xoo. This study uncovers a novel role for a primary metabolic enzyme subunit as a key regulator connecting metabolism, cell death and immunity in rice, offering potential strategies for engineering durable disease resistance.

## 4 Experimental Procedures

### 4.1 Plant Materials and Growth Conditions

The *osasa2‐1* mutant was isolated from an M_2_ population derived from tissue‐culture‐induced mutagenesis of the indica rice cultivar MH86. To facilitate map‐based cloning, an F_2_ population was established by crossing *osasa2‐1* with the indica rice cultivar 9311. The F_2_ populations and parental lines were cultivated in a summer field in Fuzhou, while the F_3_ populations and parents were grown in a winter field in Sanya. Disease and phenotype assessments were conducted by germinating sterilized rice seeds on 0.5 × Murashige and Skoog medium, followed by greenhouse cultivation under a 12‐h white light/12‐h dark photoperiod at a temperature of 28°C.

### 4.2 DAB and Trypan Blue Staining

For the measurement of hydrogen peroxide (H_2_O_2_) levels, leaves (second from the top) were harvested at the tilling stage and promptly immersed in a 1 mg/mL solution of 3,3′‐DAB before being incubated for 8 h in darkness at room temperature. Subsequently, the samples were decolourized in 95% boiling ethanol for 10 min and then soaked in 95% ethanol for 48 h to completely remove chlorophyll. The resulting cleared leaves were then photographed.

Trypan blue staining was used to detect cell death in rice leaves. Leaf samples were immersed in 65°C trypan blue solution (2.5 mg/mL trypan blue, 25% (v/v) lactic acid, 23% water‐saturated phenol, 25% (v/v) glycerol), boiled for 2 min and incubated overnight at room temperature without prior chlorophyll removal. Subsequently, leaves were destained with chloral hydrate clearing solution (2.5 g/mL in ultrapure water) as previously described (Wang et al. 2016). Stained leaves were photographed under bright‐field microscopy.

### 4.3 Measurement of ROS

The ROS burst assay described by Tian et al. (2020) was conducted in accordance with their methodology. Briefly, rice leaf discs from 7‐day‐old seedlings were cut and placed in 100 μL of water in a 96‐well plate overnight. To measure ROS production, the water was then replaced with a 100 μL reaction solution containing 20 μM luminol, 2.5 μg/mL peroxidase and 400 nM chitin (hexa‐*N*‐acetylchitohexaose). The time‐dependent quantification of ROS production was monitored using a Mithras luminometer (Berthold) at 2‐min intervals for a total of 20 min. Each sample was replicated 16 times.

### 4.4 Callose Deposition

To assess and quantify chitin‐induced callose deposition, the leaves of 7‐day‐old seedlings were excised and treated with 400 nM chitin. The experimental protocol followed previously published methods (Yang et al. 2019). Callose deposits were visualized under UV light (excitation 405 nm, emission 498 nm; Zeiss LSM880) and quantified by counting the deposits in all fields of view using ImageJ v. 1.43U software (Schneider et al. 2012).

### 4.5 Pathogen Infection

To assess the resistance of rice blast disease, leaves (second from the top) at 60 days post‐sowing were exposed to *M. oryzae* FJ‐1 using a punch method as described by Tian et al. (2020). At least three individual plants and three tillers of each plant were inoculated with one strain. Specifically, the leaves were punctured with a mouse ear punch, and a 10 μL solution of *M. oryzae* spores (5 × 10^5^ spores/mL) was administered to the wounded area, which was subsequently sealed with cellophane tape. The inoculated plants were then relocated to a growth chamber with a 12‐h light–dark cycle, a temperature of 28°C, and a relative humidity of 80%. Lesion area and fungal biomass were quantified at 12 days post‐inoculation (dpi).

Rice plants were inoculated with Xoo using the leaf‐clipping method (Liu et al. 2017). The PXO99 isolate was suspended in distilled water at a concentration of 10^9^ viable cells/mL (OD_600_ = 1). Scissors were sterilized and dipped into the bacterial suspensions before removing the distal tips (5 cm) of flag leaves. Each plant and tiller received inoculation with the strain, with a minimum of three individual plants and three tillers per plant. The infected plants were cultivated in a growth chamber under a photoperiod of 14 h of white light and 10 h of darkness at a temperature of 28°C. Disease progression was assessed by measuring lesion length at 15 dpi.

### 4.6 Map‐Based Cloning of *osasa2‐1* and Complementation

The linkage analysis initially employed a bulked segregant analysis (BSA) method, followed by the design of additional InDel markers surrounding the initial linkage locus, based on sequence polymorphism between the indica cultivars Minghui63 and 9311. Subsequently, the newly developed InDel markers were used to refine the mutation region by genotyping 420 and 610 recessive individual F_2_ and F_3_ plants, respectively. Genomic DNA fragments within the candidate region were then amplified and sequenced. To complement the *osasa2‐1* mutant, a 7468‐bp genomic fragment encompassing the entire open reading frame (ORF) of *OsASA2*, along with 2078‐bp upstream of the start codon and 1367‐bp downstream of the termination codon, was amplified via PCR using the following primers: F1‐Primer: 5′‐TGCGTTGCTGTGGTACCATCG‐3′, R1‐Primer: 5′‐TGCAAGAGTAGTGATGTCTTC‐3′; F2‐Primer: 5′‐TCTTGATCGGCATGCAACCAC‐3′, R2‐Primer: 5′‐GTGGTTGCATGCCGATCAAG. Subsequently, the PCR product was inserted into the binary vector pCAMBIA1300 using the In‐Fusion Advantage Cloning kit (Clontech). The resulting expression construct was then introduced into the *osasa2‐1* mutant via *Agrobacterium*‐mediated transformation. The CRISPR/Cas9 vector was constructed according to the method of Wang et al. (2014). The primer sequences used were gRNA: F: 5′‐GAGGCGCGAGGGCGTGAACG‐3′, R: 5′‐CGTTCACGCCCTCGCGCCTC‐3′. Two independent gRNA target sites were designed for *OsASA1*: target 1 primers (F: 5′‐GTCCAAGTTCAAGGCGGGGG‐3′; R: 5′‐CCCCCGCCTTGAACTTGGAC‐3′) and target 2 primers (F: 5′‐GTTGGGGCACAGCCTGCGA‐3′; R: 5′‐TCGCAGGCTGTGCCCCAAC‐3′).

### 4.7 RNA‐Seq Sequencing, iTRAQ Proteomic Assay and RT‐qPCR Analysis

The total RNA from MH86 and *osasa2‐1* leaves before and 48 hpi with blast fungus, and at tilling stage were extracted using the RNAeasy kit (Qiagen) and treated with an RNase‐Free DNAse Set (Qiagen), according to the manufacturer's instructions. Three biological replicates were set for each treatment. Therefore, there were 18 RNA samples in total. Each RNA sample was from a mixture of three leaf segments. The RNA samples were sequenced on Illumina HiSeq 2000 performed by Biomarker Technologies (https://www.biomarker.com.cn/, BioMarker). Raw reads were processed using fastx_toolkit_0.0.14, followed by quality control using SeqPrep and Sickle software (Table S4). TopHat2 was used for read mapping for each biological replicate in MH86 or *osasa2‐1* against the japonica cultivar Nipponbare sequence based on MSU 7.0 (Table S5, Figure S6). Cufflinks was employed for transcript assembly with reference annotation to guide the process (Trapnell et al. 2009, 2010). All procedures were conducted with predefined parameters. The identification of significant differentially expressed loci (SDEL) in the *osasa2‐1* mutant compared to MH86 was performed after implementing multiple corrections (FDR adjusted *p* ≤ 0.05). RSEM was used to quantify gene abundances. Essentially, differential expression analysis was performed using the DESeq2 or DEGseq (Li and Dewey 2011; Love et al. 2014; Wang et al. 2009). The SDEL with fold change ≥ 2 or ≤ 0.5 were used for further analysis (Figure S7). Functional enrichment analysis (FEA) was conducted on SDEL in *osasa2‐1* in comparison to MH86. A heat map was created through cluster analysis. Total protein was extracted from the leaves of the *osasa2‐1* mutant exhibiting cell death and from wild‐typeMH86 plants. The iTRAQ experiment was performed by Shanghai Applied Protein Technology Co. Ltd. as our previous report (https://www.aptbiotech.com/) (Tian et al. 2022).

Subsequently, GO and KEGG analyses were employed in the FEA to identify DEGs that exhibited significant enrichment in GO terms and metabolic pathways at a Bonferroni‐corrected *p*‐value of less than 0.05 when compared to the entire transcriptome background. GO functional enrichment and KEGG pathway analysis were carried out by Goatools and Python scipy software, respectively. The proteins data were conducted on the Majorbio I‐Sanger Cloud Platform (https://www.i‐sanger.com). Proteome Discoverer software (Thermo Fisher) was used to convert the O‐Exactive‐generated raw data into mgf files. The Mascot search engine (Matrixscience.com) was used to identify the proteins using a database containing the nonredundant protein database (NCBInr), UniProt and other databases (Figure S8). The MS/MS data were processed and expression analysis was performed using software created by Majorbio. Proteins were considered significantly differentially abundant if they had *p*‐values ≤ 0.05 (*t*‐test) and mean fold changes between *osasa2‐1* mutant and WT ≥ 1.2 or ≤ 0.83 (Figure S9). We validated the RNA level of 9 proteins through RT‐qPCR (Figure S11). The proteins identified were functionally annotated by GO database. KEGG (https://www.genome.jp/kegg/) (KOBAS 2.1.1) was used to classify and group the identified proteins. GO functional enrichment and KEGG pathway analyses were performed with Goatools (https://github.com/tanghaibao/Goatools) and KOBAS (https://kobas.cbi.pku.edu.cn/home.do) (Figure S12).

The methodology employed in this study involved conducting RT‐qPCR analysis following the protocol outlined by Wang et al. (2018). Total RNA extraction from leaves (second from the top) was carried out using TRIzol reagent (Invitrogen), followed by cDNA synthesis with a ReverTra Ace kit (Toyobo) to serve as a template for qPCR. The qPCR was conducted using a SYBR Premix ExTaq kit (TaKaRa) on a Bio‐Rad iQ2 system, with three biological replicates (three plants per replicate) and two technical replicates for each sample. Normalization of the target gene expression levels was performed relative to the *actin* gene. All experiments were conducted in triplicate. The primers used for RT‐qPCR in this study are listed in Table S6.

### 4.8 Measurements of SA, JA, JA‐Ile, ET, ABA, ACC, IAA Contents and Pathway Intermediates

Hormone levels were analysed using the MetWare platform in conjunction with the AB Sciex QTRAP 6500 LC‐MS/MS system. The third fully expanded leaf from the top was harvested at the tillering stage, and the lesion areas from both *osasa2‐1* and MH86 on the same leaf were collected for hormone quantification. Three replicates of each assay were conducted, with a 500 mg sample of powder from both the *osasa2‐1* mutant and WT plants being extracted using 5 mL of 90% aqueous methanol. Internal standards of 2 ng of each D‐labelled hormone were added to the extraction solvents for accurate content measurement. Following extraction, the crude extracts were purified using tandem MAX and MCX cartridges before being reconstituted with 200 μL of 80% methanol and analysed using UPLC‐MS/MS. The procedures adhered to the manufacturer's instructions, with hormones being detected in negative multiple reaction monitoring (MRM) mode and quantified using MRM transitions. Source parameters were configured as follows: ion spray voltage at −4500 V, temperature at 600°C, gas settings GS1 at 45, GS2 at 55 and curtain gas at 28. The quantification of hormones was conducted at the Institute of Genetics and Developmental Biology of the Chinese Academy of Sciences in Beijing, China (https://www.genetics.ac.cn/jspt/zwjs/).

Levels of pathway intermediates and derivatives in leaf parts of the *osasa2‐1* mutant exhibiting cell death and WT plants (100 mg) were quantified as previously described (Tian et al. 2020; Han et al. 2015). Reproducibility was assessed with six biological replicates in each experiment. This experiment was performed in collaboration with Shanghai Biotree Biotech Co. Ltd., Shanghai, China (https://biotreeaq.bioon.com.cn/).

### 4.9 Protein Subcellular Localization Assay

Protoplasts derived from young ZH11 etiolated rice seedlings were released and subjected to a protocol for preparation and polyethylene glycol‐mediated transfection as outlined by Trinidad et al. (2021) with slight adjustments. Following transfection, the protoplasts were resuspended in W5 buffer (154 mM NaCl, 125 mM CaCl_2_, 5 mM KCl, 5 mM glucose, 2 mM MES, pH 5.7) and subsequently incubated at 28°C for a duration of 16 h. To determine the subcellular localization of OsASA1, OsASA2, OsASB1 and OsASB2, their coding sequences were inserted into the pYBA1132 vector with a GFP tag at the C terminus. The GFP signal was observed 16 h post‐transfection using a Zeiss LSM880 confocal microscope, with excitation at 488 nm and emission at 546 nm.

### 4.10 BiFC Assay

The coding sequence of *OsASA2* was inserted into pRTVnVC, generating VcASA2, and the coding sequences of OsASA1, OsASB1 and OsASB2 were independently inserted into pRTVnVn, generating VnASA1, VnASB1 and VnASB2. The Vc‐ASA2 and VnASA1, VnASB1 and VnASB2 plasmids, were transiently coexpressed in rice protoplasts using the method described above. Yellow fluorescence (mVenus) was observed using a confocal microscope after 16 h of culturing at 28°C in the dark. Primers used in the generation of the BiFC constructs are listed in Table S6.

### 4.11 In‐Vitro AS Activity Assay

A 2.5 cm leaf of *osasa2‐1* mutant and MH86 were used for extraction. All extraction procedures were conducted at 4°C by freezing the primary leaves (approximately 0.5 g fresh weight) in liquid nitrogen and grinding them into a powder. Extraction was performed at 4°C for 10 min on a magnetic stirrer with a total of 5 mL of extract containing 100 mM Tris–HCl buffer (pH 7.5), 1 mM EDTA, 2 mM mercaptoethanol, 20 mM glutamine, 4 mM MgCl_2_ and 10% glycerol. Following filtration or centrifugation at 12,000 rpm for 10 min, the supernatant was passed through a PD‐10 column (Cytiva) equilibrated with the extract to eliminate low molecular weight compounds. The protein‐containing fractions were utilized as crude extracts for enzyme assays. The protein concentration of the extract was estimated according to Bradford (1976). A total of 0.6 mL of the substrate solution (1 μM chorismate in 0.1 M citrate/phosphate buffer, pH 7.5) was added to 0.6 mL of the extract obtained above and incubated at 30°C for 1 h. The reaction was terminated by boiling for 5 min. After centrifugation at 12,000 rpm for 10 min, it was filtered through a 0.22 μM membrane, and the filtered liquid was used for HPLC to determine the content of anthranilate. The samples were separated on a Waters XBridge C18 column (4.6 × 250 mm), and anthranilate was quantified by fluorescence detection (λex 340 nm/λem 400 nm) using 45% methanol eluting in water containing 0.1% phosphoric acid at a flow rate of 0.8 mL/min (Figure S3). In this system, anthranilate was eluted with a retention time of 7 min.

### 4.12 Split‐LUC Complementation Assay

The coding sequences of *OsASA1* and *OsASA2* were inserted into either the nLUC or cLUC vector, as described by Chen et al. (2008). The resulting constructs were subsequently co‐expressed in *N. benthamiana* leaves through *Agrobacterium*‐mediated infiltration, utilizing *Agrobacterium tumefaciens* strain GV3101. The agrobacteria were resuspended in infiltration buffer containing 10 mM MgCl_2_, 10 mM MES, 200 mM acetosyringone, at a final OD_600_ of 0.6. Three days following infiltration, detached leaves were treated with 1 mM D‐luciferin and incubated in darkness for 5–10 min. LUC activity was then measured using the LB985 NightSHADE plant imaging system (Berthold).

### 4.13 Co‐IP Assay

The co‐immunoprecipitation assays were conducted by harvesting *N. benthamiana* leaves 3 days post‐*Agrobacterium*‐mediated infiltration, followed by grinding in liquid nitrogen. Total protein was extracted using an extraction buffer containing 50 mM Tris–HCl pH 7.5, 150 mM NaCl, 1 mM EDTA, 1 mM dithiothreitol, 1% (v/v) IGEPAL CA‐630, 10% (v/v) glycerol, 1 mM PMSF and a protease inhibitor cocktail. The protein samples were then incubated with 10 μL GFP‐Nanoab‐Agarose beads (Lablead; GNA‐20‐400) at 4°C for 2 h with gentle shaking. The beads underwent four washes in washing buffer (containing 0.3% [v/v] IGEPAL CA630, equivalent to the extraction buffer) before being resuspended in 60 μL of the same washing buffer. Subsequently, the samples were subjected to separation by SDS‐PAGE and analysed via immunoblotting using anti‐GFP (TransGen, HT801, diluted at 1:1000) and anti‐HA (Abmart; 26D11, diluted at 1:5000) antibodies, along with an anti‐mouse secondary antibody (Abbkine; A21010, diluted at 1:10,000). The primer sequences used for the constructions can be found in Table S6.

### 4.14 Generation and Characterization of Transgenic Rice Lines

Several gene products including *OsASA2* were inserted into the binary vector pCAMBIA1300 using the In‐Fusion Advantage Cloning kit (Clontech). The resulting expression construct was subsequently introduced into ZH11 via *Agrobacterium*‐mediated transformation. Two target sites within *OsASA2* were selected, and the single guide RNA (sgRNA) expression cassettes were cloned into pYLCRISPR/Cas9PUbi to create the CRISPR/Cas9 vector. The constructed OE and CRISPR/Cas9 vectors were introduced into ZH11 using the *Agrobacterium*‐mediated transformation method and the homozygous overexpression and knockout mutant lines were characterised as previously described (Ma et al. 2015; Wang et al. 2021).

### 4.15 Statistical Analysis

Data are presented as mean ± standard error of mean (SE). For comparisons between two groups, Student's *t*‐test was used. For multiple group comparisons, one‐way or two‐way ANOVA was performed, followed by Tukey's honestly significant difference (HSD) post hoc test. Statistical analyses were conducted using SPSS software (v. 25.0). A *p*‐value < 0.05 was considered statistically significant.

## Author Contributions

**Mingliang Guo:** data curation, formal analysis. **Shuyu Jiang:** formal analysis, data curation. **Ying Li:** formal analysis, data curation. **Zhiwei Chen:** data curation. **Shengping Li:** supervision, methodology. **Ziqiang Chen:** data curation, formal analysis. **Yuqing Niu:** data curation. **Mo Wang:** project administration. **Songbiao Chen:** data curation, resources. **Dagang Tian:** conceptualization, project administration, investigation, funding acquisition, visualization, writing – original draft, writing – review and editing.

## Funding

This work was supported by the Public welfare research institute project (2025R1074), the National Natural Science Foundation of China (32402393, U23A20178, 32402393, 32071941 and 31301654) and External cooperation projects of FAAS (20240104).

## Conflicts of Interest

The authors declare no conflicts of interest.

## Supporting information


**Figure S1:** Cell death phenotype of *osasa2‐1* is rescued by *OsASA2*.

**Figure S2:** Subcellular localization of anthranilate synthase α‐ and β‐subunit. OsASA1‐GFP, OsASA2‐GFP, OsASB1‐GFP and OsASB2‐GFP were transient expression in rice protoplasts. The green GFP fluorescence signal was detected by confocal microscopy. Their green GFP fluorescence signal can co‐locate with red autofluorescence of chloroplast. Bar = 10 μm.

**Figure S3:** The detection map of anthranilate in Minghui86 and *osasa2‐1*.

**Figure S4:** The phenotype of *osasa1‐1*, *osasa1‐2* and Minghui86.

**Figure S5:** The length distribution of transcripts.

**Figure S6:** The statistics of significant differential expression genes. WT_mock_vs_*osasa2‐1*_mock (designated WT and *osasa2‐1* mutant mock‐treated), WT_N_vs_*osasa2‐1*_N (designated WT and *osasa2‐1* mutant under cell death) and WT_48hpi_vs_*osasa2‐1*_48hpi (designated WT and *osasa2‐1*mutant at 48 hpi).

**Figure S7:** KEGG annotation analysis of differential expressed genes in wild‐type (WT) and *osasa2‐1* mutant mock‐treated.

**Figure S8:** Pathway of those 10 genes associated with signal transduction.

**Figure S9:** The length distribution of peptide length.

**Figure S10:** The statistics of significant differential expression proteins.

**Figure S11:** Information of the 9 Homo‐directional changes of the mRNA/proteins.

**Figure S12:** GO and KEGG enrichment analysis of differential expressed proteins in the *osasa2‐1* mutant exhibiting cell death compared with wild‐type plants.

**Figure S13:** Disease phenotypes of one‐month‐old seedlings of wild‐type MH86 and the *osasa2‐1* mutants in a field rice blast nursery with natural blast infection.

**Table S1:** Comparison of agronomic traits between *osasa2‐1* and MH86.
**Table S2:** The mutation patterns of OsASA1 and OsASA2 in different lines.
**Table S3:** Hierarchical clustering heat map data.
**Table S4:** The statistics of sequencing data.
**Table S5:** The statistics of mapped.
**Table S6:** Primers for RT‐qPCR, genotyping and vector construction.

## Data Availability

RNA‐seq data from this article can be found in the GenBank/EMBL databases with the accession number CRA016648. Other data that support the findings of this study are available from the corresponding author upon reasonable request.
