# Supplementary material for: OsASA2 Loss‐of‐Function Confers Broad‐Spectrum Disease Resistance and Triggers Cell Death by Regulating Anthranilate Synthase Activity in Rice
Source: Mol Plant Pathol. 2026 Sep 6;27(9):e70340. doi: 10.1111/mpp.70340 (PMC13547970; doi:10.1111/mpp.70340)
Supplement: Supplementary file 1 [file MPP-27-e70340-s001.docx]

**Supplemental Figure S12** GO and KEGG enrichment analysis of differential expressed proteins in the *osasa2-1* mutant exhibiting cell death compared with wild-type plants.
