# Supplementary material for: OsASA2 Loss‐of‐Function Confers Broad‐Spectrum Disease Resistance and Triggers Cell Death by Regulating Anthranilate Synthase Activity in Rice
Source: Mol Plant Pathol. 2026 Sep 6;27(9):e70340. doi: 10.1111/mpp.70340 (PMC13547970; doi:10.1111/mpp.70340)
Supplement: Supplementary file 2 [file MPP-27-e70340-s002.docx]

**Supplemental Figure S4** The phenotype of *osasa1-1, osasa1-2*, and Minghui86.

(A) The greenhouse natural growth phenotype. Bar = 15 cm.
(B) Phenotypes of wild-type MH86 and the *osasa1-1* and *osasa1-2* mutants following spray inoculation with *M. oryzae* conidia. Bar = 1 cm.
(C) Relative lesion area of MH86 and *osasa1-1* and *osasa1-2* at 7 days post inoculation.
(D) Phenotype of wild type MH86 and *osasa1-1* and *osasa1-2* punched with *M. oryzae* conidia. Bar = 1 cm.
(E) Lesion length of MH86 and *osasa1-1* and *osasa1-2* at 7 days post inoculation.
(F) Relative fungal growth of MH86 and *osasa1-1* and *osasa1-2* at 48 hpi.
(G) Pathogenic phenotype of wild type MH86 and *osasa1-1* and *osasa1-2* inoculated with *Xoo*. Bar = 1 cm.
(H) Blight lesion length on leaves of *osasa1-1* and *osasa1-2* and MH86 were measured.

Data are presented as the means of three independent replicates ± SD and were statistically analyzed by one-way ANOVA, ANOVA was followed by Tukey’s honestly significant difference (HSD) post-hoc test (α = 0.05) to determine pairwise differences among groups. Bars with different letters are significantly different from each other (P < 0.05).
