# Supplementary material for: OsASA2 Loss‐of‐Function Confers Broad‐Spectrum Disease Resistance and Triggers Cell Death by Regulating Anthranilate Synthase Activity in Rice
Source: Mol Plant Pathol. 2026 Sep 6;27(9):e70340. doi: 10.1111/mpp.70340 (PMC13547970; doi:10.1111/mpp.70340)
Supplement: Supplementary file 4 [file MPP-27-e70340-s004.docx]

**Supplemental Figure S6** The statistics of significant differential expression genes

Notes: WT_mock_vs_*osasa2-1*_mock (designated WT and *osasa2-1* mutant mock-treated), WT_N_vs_*osasa2-1*_N (designated WT and *osasa2-1* mutant under cell death), and WT_48hpi_vs_*osasa2-1*_48hpi (designated WT and *osasa2-1*mutant at 48 hpi).
