# Supplementary material for: OsASA2 Loss‐of‐Function Confers Broad‐Spectrum Disease Resistance and Triggers Cell Death by Regulating Anthranilate Synthase Activity in Rice
Source: Mol Plant Pathol. 2026 Sep 6;27(9):e70340. doi: 10.1111/mpp.70340 (PMC13547970; doi:10.1111/mpp.70340)
Supplement: Supplementary file 5 [file MPP-27-e70340-s005.docx]

**Supplemental Figure S2** Subcellular localization of anthranilate synthase α and β-subunit. OsASA1-GFP, OsASA2-GFP, OsASB1-GFP and OsASB2-GFP were transient expression in rice protoplasts. The green GFP fluorescence signal was detected by confocal microscopy. Their green GFP fluorescence signal can co-locate with red autofluorescence of chloroplast. Bar = 10 μm.
