# Supplementary material for: OsASA2 Loss‐of‐Function Confers Broad‐Spectrum Disease Resistance and Triggers Cell Death by Regulating Anthranilate Synthase Activity in Rice
Source: Mol Plant Pathol. 2026 Sep 6;27(9):e70340. doi: 10.1111/mpp.70340 (PMC13547970; doi:10.1111/mpp.70340)
Supplement: Supplementary file 7 [file MPP-27-e70340-s007.docx]

**Supplemental Figure S13** Disease phenotypes of one-month-old seedlings of wild-type MH86 and the *osasa2-1* mutants in a field rice blast nursery with natural blast infection.
