# Supplementary material for: OsASA2 Loss‐of‐Function Confers Broad‐Spectrum Disease Resistance and Triggers Cell Death by Regulating Anthranilate Synthase Activity in Rice
Source: Mol Plant Pathol. 2026 Sep 6;27(9):e70340. doi: 10.1111/mpp.70340 (PMC13547970; doi:10.1111/mpp.70340)
Supplement: Supplementary file 8 [file MPP-27-e70340-s009.docx]

Minghui86-2

Minghui86-1

*osasa2-1*

Minghui86-3

*osasa2-3*

*osasa2-2*

**Supplemental Figure S3** The detection map of anthranilate in Minghui86 and *osasa2-1.*
