# Supplementary material for: OsASA2 Loss‐of‐Function Confers Broad‐Spectrum Disease Resistance and Triggers Cell Death by Regulating Anthranilate Synthase Activity in Rice
Source: Mol Plant Pathol. 2026 Sep 6;27(9):e70340. doi: 10.1111/mpp.70340 (PMC13547970; doi:10.1111/mpp.70340)
Supplement: Supplementary file 9 [file MPP-27-e70340-s010.docx]

Relative expression level

|  | iTRAQ | RT-qPCR |
| --- | --- | --- |
| Q5U1H6 | 1.46219 | 10.9655 |
| Q5VP66 | 1.39511 | 8.16659 |
| Q67VM2 | 1.33099 | 4.13886 |
| Q6ZL61 | 1.32685 | 3.19785 |
| Q75T45 | 1.40021 | 6.63463 |
| Q7F1U0 | 2.53281 | 19.3623 |
| Q8H4P7 | 1.72424 | 3.69573 |
| Q8H8C7 | 1.40984 | 2.29803 |
| Q9FTN5 | 1.3595 | 2.71776 |

**Supplemental Figure S11** Information of the 9 Homo-directional changes of the mRNA/proteins
