# Supplementary material for: OsASA2 Loss‐of‐Function Confers Broad‐Spectrum Disease Resistance and Triggers Cell Death by Regulating Anthranilate Synthase Activity in Rice
Source: Mol Plant Pathol. 2026 Sep 6;27(9):e70340. doi: 10.1111/mpp.70340 (PMC13547970; doi:10.1111/mpp.70340)
Supplement: Supplementary file 10 [file MPP-27-e70340-s011.docx]

**Supplemental Figure S7** KEGG annotation analysis of differential expressed genes in WT and *osasa2-1* mutant mock-treated
